# The Reliability of the Social Responsiveness Scale-2 in an Iranian Typically Developing Group of Children

**Published:** 2020-01

**Authors:** Zahra Shahrivar, Mehdi Tehrani-Doost, Elham Davoodi, Tanaz Hosseiniani, Helia Tarighatnia, Samane Momen, Ali Reza Sebghati, Hajirezaei Hajirezaei

**Affiliations:** 1 Department of Psychiatry, Roozbeh Hospital, Tehran University of Medical Sciences, Tehran, Iran.; 2 Research Center for Cognitive and Behavioral Sciences, Roozbeh Hospital, Tehran University of Medical Sciences, Tehran, Iran.; 3 Roozbeh Hospital, Tehran University of Medical Sciences, Tehran, Iran.

**Keywords:** *Behavior Rating Scale*, *Child*, *Social Responsiveness Scale-2*, *Validation Studies*

## Abstract

**Objective:** The Social Responsiveness Scale-2 (SRS-2) is a well-known screening instrument to assess autistic spectrum symptoms quantitatively. This study assessed the different types of reliability of the Farsi translation of the scale.

**Method** **:** This scale was translated into Farsi and back translated considering all aspects of methodology in translation. Then, based on stratified sampling, 533 7-11-year-old students were randomly selected from the mainstream schools located in the central parts of Tehran, the capital of Iran. For all the students, SRS-2 was completed by both the parents and teachers. To check retest reliability, the test was administered again for 15% of the total participants after a 2-4 week-period. Cronbach’s alpha coefficient, split-half” and Gottman” methods, and intra-class correlation coefficient (ICC) were used.

**Results: **The mean total raw score was 48.47 (±23.63) and 53.17 (±27.33) in the sequence of the parents and teachers’ reports. The internal consistency (Cronbach’s alpha; 0.86 and 0.89), test-retest reliability (ICC; 0.72 and 0.83 for parents and teacher’ ratings, respectively), and interrater reliability (ICC; 0.72) showed well-accepted measurement performance.

**Conclusion: **The findings indicated that the Farsi SRS-2 can be used as a reliable instrument to measure social responsiveness as autistic symptoms in Iranian child population.

Autism spectrum disorder is one of the major neurodevelopmental disorders characterized by social communication deficits and repetitive behavior /restricted interests and repetitive and restricted patterns of behavior and interests (1). Some instruments have been developed to screen these symptoms in normal and clinical population. Among these tools, the most popular are Modified Checklist for Autism in Toddlers (M-CHAT) (2), Pervasive Developmental Disorders Screening Test, Second Edition (PDDST-II) (3), Social Communication Questionnaire (SCQ) (4), and Social Responsiveness Scale (SRS) (5). The M-CHAT is limited to the toddler period, the PDDST-II is based on DSM-IV, and the SCQ has been mostly used in clinical population. Compared to these tools, SRS can be used from childhood to adult age, is based on DSM-5 classification, and can be administered to community samples. Moreover, the Social Responsiveness Scale-2 (SRS-2) is an instrument being used to measure autistic traits in children, adolescents, and adults (6). It characterizes a quantitative assessment of the impairment and severity in social interaction and communication and repetitive behavior /restricted interests as the main characteristics of the autism spectrum disorder (7). In addition to being used in children at risk or diagnosed as autistic spectrum, SRS can be used in large general populations to measure mild problems in social interaction and communication. Because of these characteristics, SRS was implemented in Farsi speaking community.

SRS was originally developed by Constantino & Todd, 2003 (7), and revised by Constantino and Gruber, 2012 (8). The psychometric properties of SRS have been reported as good (4-6). Several studies have used SRS to compare the autistic symptoms in clinical and nonclinical groups of children (9 & 10). However, as other Language versions of a measure may be performed differently from the original English version, some studies provided the normative properties of SRS in other countries or cultures; for example, the Spanish (9), German (11), and Chinese (12) versions. 

SRS can help to find and scale the autistic symptoms as a spectrum in general population of children. Detection of the at-risk population can help the parents to seek early intervention. Hence, translation and validation of the test for the Iranian community can help mental health professionals to approach to these children. In this study, it was aimed to assess the psychometrics of the Farsi translation of the test in a group of school-aged children in Tehran, the capital of Iran. The validity of the Farsi version of the test was acceptable; the findings have been reported elsewhere (see procedure and methods section). This paper is a part of the larger study which presents the internal consistency, interrater, and test-retest reliabilities of the test. To our knowledge, no study has been published on the reliability properties of SRS in Farsi speaking children. Thus, it was hypothesized that the Farsi translation of SRS is reliable to screen subtle symptoms of social communication deficits and repetitive behavior / restricted interests as autistic traits in Iranian elementary school students.

## Materials and Methods

***Participants***

The total number of 533 students were recruited in the study. Since it was needed to assess the mean scores of SRS in the target population, the sample size was calculated based on the n = Z21-α/2 S2/ d2 formula. Considering Z = 1.96, S = 21 (ref), and d = 2, the derived size (1.962 x 212/22) is equal to 423. Regarding the probable 20% attrition, the sample was increased to 533 (13 & 14).

The participants were parents and teachers of 342 girls and 191 boys aged 7-11 years studying at grade 1 to 6 in 4 central regions of Tehran. Distribution of the students among the 6 academic levels was approximately the same (17% in each grade of 1, 2, 3, 4; 15% in each grade of 5 and 6). 

***Procedure and Measure***

The study had a cross sectional random clustering design and participants were recruited from September 2016 to March 2017. The central regions were selected based on the fact that the students’ demographic characteristics in these parts were representative of the middle-class socioeconomic population living in the capital. Parents of the students in 32 elementary schools (8 schools in each region) were invited to take part in the study by a home message accredited by the school principal. Of these schools, 3 did not cooperate at all, 1 was replaced by another school, and 2 were removed from the study. One school due to the preponderance of Afghan students and another due to Armenian children were removed as well. In 2 other schools, the Afghan students were not included in the study. After the parents consented to participate in the study, they (all were mothers) attended the school where their children where studying and were asked to complete the demographic questionnaire as well as SRS. Among the 939 parents invited, 616 individuals (65%) attended the school meetings. The response rate among the teachers (86%) was higher than the parents (57%). The psychologists gathering the research data were available at the meeting to introduce the tool and provide explanations and answer questions. Teachers of the same students completed SRS. Finally, 533 questionnaires were thoroughly completed to be analyzed. 

Among the parents and teachers who accepted to participate in the second stage of the study, 15% were randomly invited to rate SRS again after 14-28 days. Also, 86% of the teachers and 49% of the parents completed SRS.

This study was approved by the Ethics’ Committee of Tehran University of Medical Sciences and was a part of a research funded under the grant number of 93-03-30-24758.

***Social Responsiveness Scale-2 (SRS-2)***

The process of translation of the scale (15) from English to Farsi, back translation, and the pilot evaluation of the scale, as well as its validation data have been described thoroughly elsewhere (16). The scale has 65 items and can be completed by parents, teachers, or any caregiver who knows the child. SRS can be easily answered in 15-20 minutes. The respondents rate the items in an ordinal-scaled method from 1(not true) to 4 (almost always true). The raw scores of the scale produce 2 principal subscales of “Restricted Interests & Behavior” and “Social Communication & Interaction”. The latter consists of 4 subscales, including social awareness, social cognition, social communication, and social motivation.

***Statistical analysis***

The SPSS (version 18) was used to perform the statistics. Descriptive method was conducted to assess the means and standard deviations. The independent t test and chi-square were used to compare the data based on age and gender, respectively. Internal consistency was calculated using the Cronbach’s alpha coefficient. Split-half” and “the Gottman” methods were used to test internal reliability. Interrater and test-retest reliability were assessed by intraclass correlation coefficient (ICC).

## Results

The demographic characteristics of the participants are presented in Table 1. There were no significant differences between the girls and boys based on their mean ages. 

The means (±standard deviations) and medians for the parents and teachers’ reported scores of SRS scales are presented in Table 2. No significant differences were found in SRS mean scores in terms of gender and age (details have been explained elsewhere, under review).

To evaluate the internal consistency of SRS, Cronbach’s alpha was generated for SRS total raw score as well as the scales. Based on the parents’ and teachers’ reports respectively, Cronbach’s alpha was 0.07 and 0.31 for the “social awareness”, 0.65 and 0.62 for “social cognition”, 0.83 and 0.86 for the “social communication”, 0.63 and 0.65 for the “social motivation”, 0.80 and 0.88 for the “restricted interests & behavior”, and 0.86 and 0.89 for the “total scores”. Besides, the split-half internal reliability using the Spearman Brown formula showed excellent results (0.87 for parents and 0.90 for teachers’ reports). Also, the Gottman coefficient was calculated and confirmed the internal consistency of SRS (0.87 and 0.90 for the results by parents and teachers, respectively) (8).

The inter-rater reliability among the parents and teachers was calculated using the intra-class correlation coefficient (ICC) (Table 3).

The test-retest reliability intraclass correlation coefficient for SRS based on the parents and teachers’ reports has been demonstrated in table 4.

**Table 1:** Demographic Characteristics of the Children for the Two Separate Stages of the Study

**First stage of the study** **Total (N=533)**		**Second stage of the study** **Total (N=69)**	
**Girls (N=342)** **Mean age=9.50 (±1.74)**	**Boys (N=191)** **Mean age=9.40 (±1.68)**	**Girls (N=42)** **Mean age=9.54 (±1.90)**	**Boys (N=27)** **Mean age=9.62 (±1.59)**
Academic level Number (%)	City region Number (%)	Academic level Number (%)	City region Number (%)
First: 92 (17%)	6: 70 (13%)	First: 12 (17.4%)	6: 5 (7.2%)
Second: 91 (17%)	7: 180 (33%)	Second: 10 (14.5%)	7: 23 (33.3%)
Third: 93 (17%)	11: 130 (24%)	Third: 13 (18.8%)	11: 20 (29%)
Fourth: 95 (17%)	12: 153 (28%)	Fourth: 10 (14.5%)	12: 21 (30.4%)
Fifth: 82 (15%)		Fifth: 11 (15.9%)	
Sixth: 80 (15%)		Sixth: 13 (18.8%)	

**Table 2:** The SRS Scales Scores Means (±SD) and Median of the Children in the First Stage of the Study Based on the Parents and Teachers’ Reports Separately

**SRS Scales**	**Parents’ report (N=533)**		**Teachers’ report (N=533)**	
	**Mean ± SD**	**Median**	**Mean ± SD**	**Median**
Social Awareness	8.25 ± 2.79	8	9.06±3.30	9.00
Social Cognition	8.98 ± 4.94	8	10.01±5.36	9.00
Social Communication	14.03 ± 9.12	12	16.99±10.18	15.00
Social Motivation	9.56 ± 4.92	9	9.57±5.16	9.00
Restricted Interests & Behavior	7.61 ± 5.91	6	7.46±6.88	6.00
Social Communication & Interaction	40.85 ± 18.76	37.98	45.66±21.35	43.00
Total Score	48.47 ± 23.63	43	53.17±27.33	47.64

**Table 3:** Inter-Rater Reliability Intra-Class Correlation Coefficients (ICC) based on Parents and Teachers’ Reported SRS Scales Mean Scores for Children

**SRS Scales**	**ICC**	**F**	**df1**	**df2**	**P**
Social Awareness	0.179	1.22	532	532	0.009
Social Cognition	0.219	1.28	532	532	0.002
Social Communication	0.252	1.35	531	531	0.000
Social Motivation	0.291	1.40	531	531	0.000
Restricted Interests & Behavior	0.284	1.39	530	530	0.000
Social Communication & Interaction	0.274	1.39	530	530	0.000
Total Scores	0.72	1.38	529	529	0.000

ICC: Intraclass Correlation Coefficient, Confidence interval = 95%

SRS-2: Social Responsiveness Scale-2

**Table 4:** Test-Retest Reliability Intra-Class Correlation Coefficient (ICC) for Parents and Teachers’ Reported SRS Mean Scores

**SRS Scales** **Parents/Teachers’ reports**	**ICC**	**F**	**df1**	**df2**	**P**
Social Awareness	0.495/0.784	2.008/4.774	68/68	68/68	0.002/0.000
Social Cognition	0.589/0.726	2.436/3.619	68/68	68/68	0.000/0.000
Social Communication	0.664/0.821	3.039/5.082	68/67	6867	0.000/0.000
Social Motivation	0.796/0.821	4.927/5.541	68/67	68/67	0.000/0.000
Restricted Interests & Behavior	0.683/0.759	3.191/4.147	68/67	68/67	0.000/0.000
Social Communication & Interaction	0.714/0.840	1.39	68/67	68/67	0.000/0.000
Total Scores	0.721/0.838	3.680/6.130	68/67	68/67	0.000/0.000

SRS-2: Social Responsiveness Scale-2

## Discussion

This study aimed to provide the reliability of the social responsive scale (SRS) in a population of school-aged children recruited from elementary schools located in 4 central regions of Tehran. To our best knowledge, this was the first study to use a Farsi translation of SRS in a community sample.

The study confirmed the internal reliability of SRS based on both the parents and teachers’ ratings (Cronbach’s Alfa: 0.85 and 0.94). These results replicated the data derived from previous studies in the US (17), the UK (18), Germany (11), Mexico (9), Taiwan (5), China (19), and South Korea (20), which reported the Alfa from 0.80 to 0.97 for the total raw mean score of SRS. Using the split-half method, a recent study provided more evidence supporting the internal consistency of the test, based on the reports from both informants. With regards to the different scales of SRS, the above-mentioned studies reported that the lowest reliability coefficient belonged to social awareness and the highest to social communication (17, 18, 19, 21 & 22). It was suggested that the different reliability coefficients among these scales can be explained by the number of related items in SRS. The social awareness domain has the lowest items in relation to the other domains of SRS. Moreover, it seems that understanding of this field may be more difficult for the respondents, especially for those with lower education. In line with the above-mentioned studies, the internal consistency of SRS among Iranian students was higher than the total score compared to the scores of each SRS scale, with the lowest Alfa calculated for social awareness. Altogether, the findings of the different studies in Western and Eastern countries showgood to excellent coherence within the full scale (especially for the clinical groups) (21).

The findings of the Iranian population showed a correlation coefficient between the parents and teachers which was satisfactory (0.7). Although the mean scores of the teachers’ answers were higher than that reported by parents, they were significantly correlated. This difference can be explained by the fact that teachers have more opportunities to observe children in social situations and judge about their social behavior. The interrater reliability of SRS has been reported as acceptable by most studies. Constantino & Gruber (6) showed that the correlation coefficient between mothers and fathers was 0.91, and the mothers and fathers’ reports were correlated with teachers’ ratings in the sequence of 0.82 and 0.75. They suggested that mothers may recognize internalizing symptoms in their children better than the fathers. The correlation coefficient between German mothers and fathers (11) was reported as 0.61-0.76, while it was 0.75 for a group of preschoolers with and without ASD (15). Fombone et al (9) found a correlation strength of 0.49 between Mexican parents and teachers, which was significantly lower in normal children (0.18) compared to the group with autism psychopathology (0.50). The ICC between the parents and teachers for all the Japanese children with and without ASD was 0.48 (0.50 for girls and 0.40 for boys) (23). A study with small sample size was conducted on Japanese children with and without ASD; and teachers rated non-significantly higher scores for both typically developing girls and boys compared to parents, while it was the opposite for children with ASD.

Similar to the internal consistency findings, some studies suggest a better reliability between teachers and parents in rating SRS items when it comes to a clinical population of children. It seems that in the continuum of social communication problems distributed in the community, recognizing the symptoms may be difficult for the informants due to the ceiling effect while recognizing the higher degrees of psychopathology; however, when it comes to children with ASD, it is easier (9) for both parents and teachers. 

The test-retest reliability of the Farsi translation of SRS was acceptable to good for the parents’ reports and it was good to excellent for the reports by teachers. These results which are consistent with the data from the above-mentioned studies in different countries confirm the ideal test-retest reliability for SRS (from 0.72 to 0.97 based on different raters and examinees). Cen et al (19) calculated the coefficient equal to 0.96 for SRS total raw score after a 2 week-interval, while Kamio et al (23) reported a kappa coefficient of 0.87 between the ratings of SRS in a much longer period (12-131 days). These findings support the reliability of SRS; and it can be used as an outcome measure to evaluate the quantitative changes in social communication, repetitive behavior, and restricted interests in response to treatment or other interventions.

To sum up, this study showed good to excellent reliability for the Farsi translation of SRS, which is in line with the studies conducted in other countries with different translations of the scale. Therefore, SRS can be used to quantify autistic traits in Iranian school-aged children with the similar characteristic of the population participating in the recent study.

## Limitation

To our knowledge, this was the first study to evaluate the reliability of the Farsi translation of SRS. As its strengths, the study assessed both teachers and parents’ reports within a rather large sample of typically developing children recruited among the general population. However, the results of this study should be considered in light of some limitations: the fathers did not participate the study, the students were mostly girls, and the schools were located in the central parts of Tehran, the capital of Iran. Hence, a generalization of the findings to other Farsi-speaking children should be considered with caution. Moreover, there were 2 groups of respondents, including teachers and parents. Although the settings for these respondents were different, the parents completed the scales in the groups while the teachers were free to answer the test based on their schedule. To lower the probable situational difference, the research assistants were available to both groups and monitored the forms to be completely answered. Another issue was the rather lower response rate of the parents compared to the teachers’, which might have led to some selection bias of the sample.

## Conclusion

This study provided strong evidence for the Farsi version of SRS to be used as a reliable test to screen school-aged children with problems in social interaction and communication, as well as restricted and stereotyped interests and behavior. However, further studies are suggested to evaluate the test characteristics in other age groups and in clinical groups of children, including those with ASD, compared to those with other neurodevelopmental disorders.

## References

[1] Association AP (2013). Diagnostic and statistical manual of mental disorders. BMC Med.

[2] Robins DL, Fein D, Barton ML, Green JA (2001). The Modified Checklist for Autism in Toddlers: an initial study investigating the early detection of autism and pervasive developmental disorders. J Autism Dev Disord.

[3] Baird G, Charman T, Cox A, Baron-Cohen S, Swettenham J, Wheelwright S (2001). Screening and surveillance for autism and pervasive developmental disorders. Arch Dis Child.

[4] Filipek PA, Accardo P, Ashwal S, Baranek G, Cook E, Dawson G (2000). Practice parameter: screening and diagnosis of autism: report of the Quality Standards Subcommittee of the American Academy of Neurology and the Child Neurology Society. Neurology.

[5] Rutter M, Bailey A, Lord C (2003).

[6] Constantino JN, Gruber CP (2005). Social Responsiveness Scale (SRS).

[7] Constantino JN, Todd RD (2003). Autistic traits in the general population: a twin study. Arch Gen Psychiatry.

[8] Constantino J N, Gruber C P (2012). Test review: “Social Responsiveness Scale”.

[9] Fombonne E, Marcin C, Bruno R, Tinoco CM, Marquez CD (2012). Screening for Autism in M exico. Autism Research.

[10] Wong VC, Hui SL (2008). Epidemiological study of autism spectrum disorder in China. Journal of Child Neurology.

[11] Bölte S, Poustka F, Constantino JN (2008). Assessing autistic traits: cross‐cultural validation of the social responsiveness scale (SRS). Autism Res.

[12] Gau SS-F, Liu L-T, Wu Y-Y, Chiu Y-N, Tsai W-C (2013). Psychometric properties of the Chinese version of the social responsiveness scale. Res Autism Spectr Disord.

[13] Constantino JN, Todd RD (2003). Autistic traits in the general population: a twin study. Arch Gen Psychiatry.

[14] Bölte S, Poustka F, Constantino JN (2008). Assessing autistic traits: cross‐cultural validation of the social responsiveness scale (SRS). Autism Res.

[15] Pine E, Luby J, Abbacchi A, Constantino JN (2006). Quantitative assessment of autistic symptomatology in preschoolers. Autism.

[16] Tehrani-Doost M, Shahrivar Z, Torabi N, Ansari S, Haji-Esmaeelzadeh M, Saeed-Ahmadi S (2018). Cross-Cultural Validation and Normative Data of the Social Responsiveness Scale in a Group of Iranian General Child Population. J Autism Dev Disord.

[17] Constantino JN, Davis SA, Todd RD, Schindler MK, Gross MM, Brophy SL (2003). Validation of a brief quantitative measure of autistic traits: comparison of the social responsiveness scale with the autism diagnostic interview-revised. Journal of autism and developmental disorders.

[18] Wigham S, Mc Conachie H, Tandos J, Le Couteur AS (2012). The reliability and validity of the Social Responsiveness Scale in a UK general child population. Res Dev Disabil.

[19] Cen C-Q, Liang Y-Y, Chen Q-R, Chen K-Y, Deng H-Z, Chen B-Y (2017). Investigating the validation of the Chinese Mandarin version of the Social Responsiveness Scale in a Mainland China child population. BMC psychiatry.

[20] Cheon K-A, Park J-I, Koh Y-J, Song J, Hong H-J, Kim Y-K (2016). The social responsiveness scale in relation to DSM IV and DSM5 ASD in Korean children. Autism Res.

[21] Wang J, Lee L-C, Chen Y-S, Hsu J-W (2012). Assessing autistic traits in a Taiwan preschool population: cross-cultural validation of the Social Responsiveness Scale (SRS). J Autism Dev Disord.

[22] Duku E, Vaillancourt T, Szatmari P, Georgiades S, Zwaigenbaum L, Smith IM (2013). Investigating the measurement properties of the social responsiveness scale in preschool children with autism spectrum disorders. J Autism Dev Disord.

[23] Kamio Y, Moriwaki A, Inada N (2013). Utility of teacher-report assessments of autistic severity in Japanese school children. Autism Res Treat.

